# Characterization of Breast Tumors Using Diffusion Kurtosis Imaging (DKI)

**DOI:** 10.1371/journal.pone.0113240

**Published:** 2014-11-18

**Authors:** Dongmei Wu, Guanwu Li, Junxiang Zhang, Shixing Chang, Jiani Hu, Yongming Dai

**Affiliations:** 1 Shanghai Key Laboratory of Magnetic Resonance, East China Normal University, Shanghai, China; 2 Department of Radiology, Wayne State University, Detroit, Michigan, United States of America; 3 Department of Radiology, Yue Yang Hospital of Integrated Traditional Chinese and Western Medicine, Shanghai University of Traditional Chinese Medicine, Shanghai, China; 4 Department of Radiology, The First Affiliated Hospital of Bengbu Medical College, Bengbu, Anhui Province, China; 5 Philips Healthcare, Shanghai, China; University Medical Center Utrecht, Netherlands

## Abstract

**Aim:**

The aim of this study was to investigate and evaluate the role of magnetic resonance (MR) diffusion kurtosis imaging (DKI) in characterizing breast lesions.

**Materials and Methods:**

One hundred and twenty-four lesions in 103 patients (mean age: 57±14 years) were evaluated by MR DKI performed with 7 b-values of 0, 250, 500, 750, 1,000, 1,500, 2,000 s/mm^2^ and dynamic contrast-enhanced (DCE) MR imaging. Breast lesions were histologically characterized and DKI related parameters—mean diffusivity (MD) and mean kurtosis (MK)—were measured. The MD and MK in normal fibroglandular breast tissue, benign and malignant lesions were compared by One-way analysis of variance (ANOVA) with Tukey's multiple comparison test. Receiver operating characteristic (ROC) analysis was performed to assess the sensitivity and specificity of MD and MK in the diagnosis of breast lesions.

**Results:**

The benign lesions (n = 42) and malignant lesions (n = 82) had mean diameters of 11.4±3.4 mm and 35.8±20.1 mm, respectively. The MK for malignant lesions (0.88±0.17) was significantly higher than that for benign lesions (0.47±0.14) (*P*<0.001), and, in contrast, MD for benign lesions (1.97±0.35 (10^−3^ mm^2^/s)) was higher than that for malignant lesions (1.20±0.31 (10^−3^ mm^2^/s)) (*P*<0.001). At a cutoff MD/MK 1.58 (10^−3^ mm^2^/s)/0.69, sensitivity and specificity of MD/MK for the diagnosis of malignant were 79.3%/84.2% and 92.9%/92.9%, respectively. The area under the curve (AUC) is 0.86/0.92 for MD/MK.

**Conclusions:**

DKI could provide valuable information on the diffusion properties related to tumor microenvironment and increase diagnostic confidence of breast tumors.

## Introduction

Breast MRI has been increasingly used in diagnosing patients with suspicious breast lesions owing to its non-invasive nature and excellent soft-tissue contrast as compared with other diagnostic imaging modalities [1]–[4]. In particular, diffusion MRI is a non-invasive MR imaging technique that allows in vivo characterization and quantification of the molecular water diffusion in tissues [5]–[7]. Specifically, measurement of functional parameters that reveal the water diffusion in microscopic environment can serve to characterize the pathological conditions of breast lesions [8]. For instance, mean diffusivity (MD) is a measure of the water diffusivity in diffusion-weighted imaging (DWI). The measurement of such water diffusion rate has been shown to help distinguish between normal fibroglandular breast tissue and benign or malignant lesions [9]–[17]. However, benign lesions and malignant cancers cannot always be discriminated accurately from each other because of the confounding overlap in their diffusion rate values. As such, advanced diffusion MR imaging techniques which may provide better characterization of breast tumor tissues are highly desirable to assist the classification and differential diagnosis of breast lesions.

Tumor invasiveness (grade and aggressiveness) is highly related to the pathophysiologic features of tumor tissues such as cytological patterns or organization, cellular structure and density [18], which are indirectly reflected in molecular diffusion properties of water molecules. Conventional diffusion MRI techniques always assume a Gaussian diffusion (i.e., free and unrestricted diffusion) of water protons. In fact, water diffusion in the complex biological tissues has a non-Gaussian distribution of water displacement profile in the presence of diverse barriers and compartmentalization that restrict the free displacement of water molecules, instead of a simplified Gaussian probability density function [19]–[23]. Indeed, non-mono-exponential diffusion-weighted (DW) signal decay has been observed with high b-values, likely due to restricted water diffusion associated with the underlying microstructures [23]–[26]. Accordingly, measurement of diffusional non-Gaussianity (i.e., diffusion kurtosis), a measure of diffusional heterogeneity, by means of diffusion kurtosis imaging (DKI) may allow improved characterization of water diffusion properties in the tumor microenvironment.

Recently, DKI parameters have been proved to be able to indicate microstructural changes within cerebral glioma tissue that affect the way that molecule diffuse and to allow better differentiation among cerebral glioma grades than those of conventional DWI [27], [28]. Moreover, the applications of DKI in characterization of the non-gaussian diffusion behavior have been successfully extended from cerebral gliomas to hepatic fibrosis [29], hepatic carcinoma [30] and prostate cancer [31]. In this study, we hypothesize that DKI could provide additional information about the water diffusion in the breast tumor microenvironment as compared with the conventional DWI. The aim of this study was to investigate and evaluate the role of DKI in characterizing breast lesions by examining the relationship between DKI parameters and tumor types in human patients with breast tumors.

## Materials and Methods

### Ethics Statement

All research procedures were approved by our institutional review boards (Yue Yang Hospital of Integrated Traditional Chinese and Western Medicine and The First Affiliated Hospital of Bengbu Medical College). Written informed consent for each study was obtained independently for all patients.

### Study Population and Pathological Examination

In total, 103 female patients (mean age  = 57±14 years; age range  = 24 to 87 years) with suspicious breast lesions (*N* = 124) were included in this study from September 2012 to June 2014. Following breast MRI, needle or excision biopsies were performed on patients to obtain lesion samples. All biopsy specimens were fixed in 10% formalin and embedded in paraffin. The specimens were then cut and stained with hematoxylin and eosin (H&E), followed by histological examination. The lesion classification based on pathological analysis of biopsy specimens was considered as standard reference. Each breast lesion was assigned as either benign or malignant. There were 42 benign lesions in 30 patients, including 17 fibroadenomas, 9 fibrocystic changes, 16 cysts; and 82 malignant lesions in 73 patients, including 27 infiltrating ductal carcinomas, 21 ductal carcinomas in situ, 23 infiltrating lobular carcinomas, 11 lobular carcinoma in situ.

### Breast MR Imaging

Bilateral breast MR imaging was performed on two 3.0 Tesla MR systems (MAGNETOM Verio; Siemens Medical Solutions, Erlangen, Germany; Achieva TX, Philips Healthcare, Best, The Netherlands) with a maximum gradient strength of 45(40) mT/m and a maximum slew rate of 200 T/m/s. 46 patients (mean age  = 59±14 years; age range  = 31 to 87 years) were scanned in a prone position on Siemens scanner with a dedicated phased-array 4-channel bilateral breast coil; while 57 patients (mean age  = 55±13 years; age range  = 24 to 79 years) were scanned in prone position on Philips scanner with a dedicated phased-array 7-channel bilateral breast coil. T2-weighted images were first acquired with interleaved multi-slice turbo spin-echo (TSE) sequence with repetition time (TR)  = 3000 ms, echo time (TE)  = 61 ms, Inversion Time (TI)  = 230 ms, turbo factor  = 16, field of view (FOV)  = 320×320 mm^2^, acquisition matrix size  = 320×320, slice thickness  = 4.5 mm, number of slices  = 30, parallel imaging acceleration factor  = 2 using generalized autocalibrating partially parallel acquisition (GRAPPA, Siemens) or Sensitivity Encoding (SENSE, Philips), number of averages (NA)  = 2, and acquisition time  = 2 minutes. For DKI, interleaved multi-slice DWI was performed using a single-shot spin-echo echo planar imaging (EPI) sequence with 7 b-values of 0, 250, 500, 750, 1000, 1500 and 2000 s/mm^2^ in x, y and z directions with TR  = 4000 ms, TE  = 100 ms, receiver bandwidth  = 1184 Hz/Pix, FOV  = 166×380 mm^2^, acquisition matrix  = 84×192, slice thickness  = 7.0 mm, number of slices  = 12, parallel imaging acceleration factor  = 2 using GRAPPA or SENSE, fat suppression achieved by spectral adiabatic inversion recovery (SPAIR), “distortion correction”  =  Yes, NA  = 1, and acquisition time  = 2.7 minutes. Dynamic contrast-enhanced T1-weighted MRI was then performed using fat-saturated three-dimensional (3D) fast low-angle shot (FLASH) sequence with TR  = 4.73 ms, TE  = 1.71 ms, flip angle (FA)  = 10°, FOV  = 320×320 mm^2^, acquisition matrix  = 384×384, slice thickness  = 1.2 mm, number of slices  = 128, parallel imaging acceleration factor  = 2 using GRAPPA or SENSE, NA  = 1, and acquisition time  = 50 sec for each time point, before (pre-contrast) and five times after injection (post-contrast) of gadolinium chelate (Magnevist; Bayer Healthcare, Berlin, Germany) at a dose of 0.1 mmol/kg at a rate of 2 mL/s followed by a flush of saline solution.

### Image and Data Analysis

MR images were analyzed in MATLAB (MathWorks, Natick, MA). Apparent diffusion coefficient (ADC) and apparent kurtosis coefficient (AKC) along each diffusion gradient direction were derived per voxel simultaneously by least-square fitting the DW signals non-linearly to: 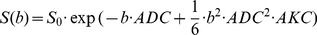
(1) where S(b) is the DW signal at a particular b-value and S_0_ is the signal without diffusion weighting. Note that we excluded the b0 (i.e., b-value  = 0 s/mm^2^) images from the numerical fitting to minimize blood perfusion effects on the DW signal decay. Background voxels with signal intensities below a threshold value in the b0 images were excluded from the fitting to increase efficiency. MD and MK maps were then calculated as the average of the parametric ADC and AKC maps along all applied diffusion gradient directions, respectively [23]. Contrast-enhancement maps were calculated as the subtraction images of the post-contrast and pre-contrast dynamic contrast-enhanced T1-weighted images. Contrast-enhancement curve (percentage signal intensity enhancement versus image frame) was also evaluated. Region of interest (ROI) analysis was performed manually by delineating breast lesions on the DW b1000 (b-value  = 1000 s/mm^2^) images with reference to the contrast-enhanced T1-weighted images. Care was taken to avoid region's cyst and necrosis that were hyperintense on both T2W images and MD maps, as well as regions of adipose tissue that were hypointense on b0 image. ROI with similar size was also defined in normal fibroglandular breast tissue in the contralateral breast. Lesion and normal fibroglandular breast tissue ROIs were then transferred and used for MD and MK measurements on the MD and MK maps. Lesion sizes were defined as the longest dimension on DCE-MRI. Results were expressed as mean ± standard deviation (SD). Prior to the analysis of DKI parameters for lesions, the study performed on two different scanners necessitates a pilot analysis to evaluate the vendor-specific differences of DKI. An independent samples t-test was used to compare DKI parameters for the ROIs of normal fibroglandular breast tissue, which were divided into two groups according to the scanners. Then, One-way analysis of variance (ANOVA) with Tukey's multiple comparison test was performed to compare the MD and MK values among normal fibroglandular breast tissue, benign and malignant tissues. A *P*-value of less than 0.05 was considered statistically significant. In addition, receiver operating characteristic (ROC) analysis was carried out to determine suitable MD and MK cutoff values for discrimination between benign and malignant lesions and to assess the diagnostic performance of DKI.

## Results

57 ROIs of normal fibroglandular breast tissue (31.5±23.7 mm, range  = 7–86 mm) obtained on Siemens scanner had mean values of 2.22±0.41 (10^−3^ mm^2^/s) and 0.55±0.15 for MD and MK respectively, while 67 ROIs of normal fibroglandular breast tissue (24.2±10.2 mm, range  = 11–70 mm) obtained on Philips scanner had mean values of 2.14±0.36 (10^−3^ mm^2^/s) and 0.53±0.14 for MD and MK respectively. No significant differences were found for MD (*P* = 0.23) and MK (*P* = 0.54) from these two groups.

**Figure 1:**
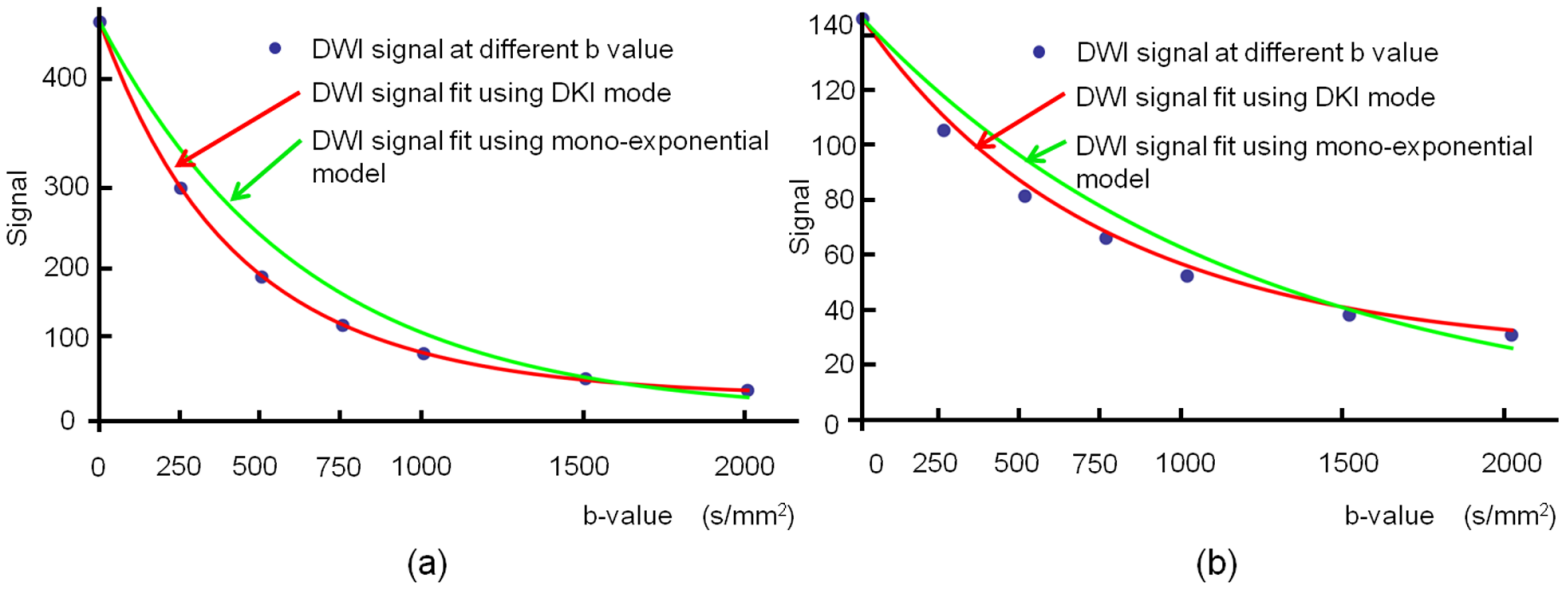
DW signal decay of breast lesion at 7 b-values of 0, 250, 500, 750, 1000, 1500 and 2000 s/mm^2^ was fitted using mono-exponential model and DKI model: a) a 59 year-in-old woman with fibroadenomas and b) a 43 year-in-old woman with ductal carcinomas in situ.

Table 1 summarizes the breast tumor types in the patient population. The mean sizes of benign and malignant lesions were 11.4±3.4 mm (range  = 7–18 mm) and 35.8±20.1 mm (range  = 11–87 mm), respectively. Figure 1 shows the DW signal decay of breast lesion in two patients with benign and malignant lesions separately, along with the fitted curve using mono-exponential model and DKI model (Eq. 1).

**Table 1:** Tumor types confirmed by pathological examination.

Benign	Malignant
FAs (n = 17, 11.8±3.1 mm)	IDC (n = 27, 37.3±22.8 mm)
FC (n = 9, 9.6±2.3 mm)	DCIS (n = 21, 29.0±9.0 mm)
Cysts (n = 16, 12.1±3.9 mm)	ILC (n = 23, 37.8±21.1 mm)
	LCIS (n = 11, 39.8±25.7 mm)

Note. – FC  =  fibrocystic changes; FAs  =  fibroadenomas;

DCIS  =  ductal carcinomas in situ; IDC  =  infiltrating ductal carcinomas;

LCIS  =  lobular carcinoma in situ; ILC  =  infiltrating lobular carcinoma.

**Figure 2:**
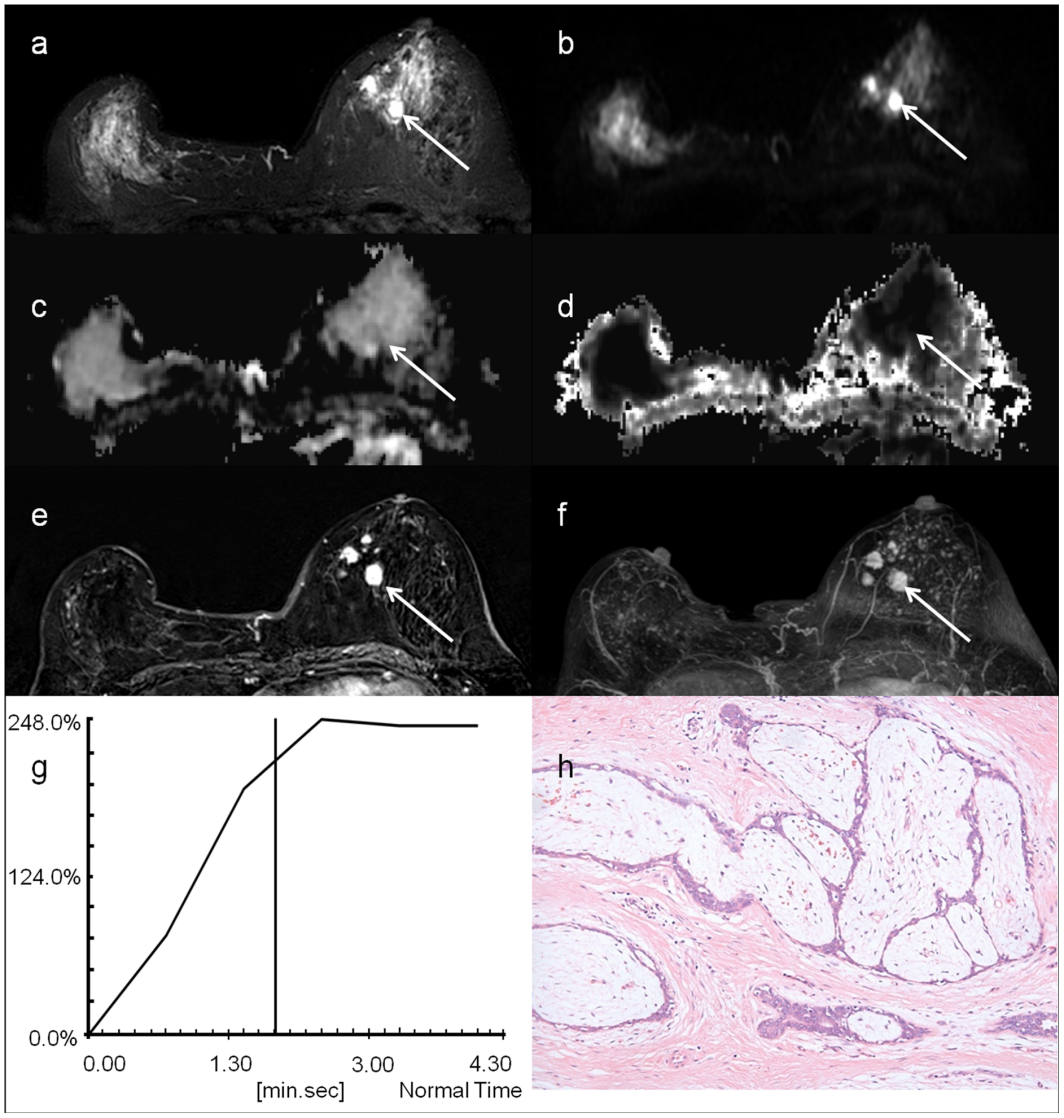
A 56 year-in-old woman with fibroadenomas, indicated by the white arrow: a) T2-weighted TSE image; b) DW image at b = 0; c) MD map; d) MK map; e) contrast enhancement map; f) MIP of enhance map; g) contrast enhancement curves and h) histological specimen.

**Figure 3:**
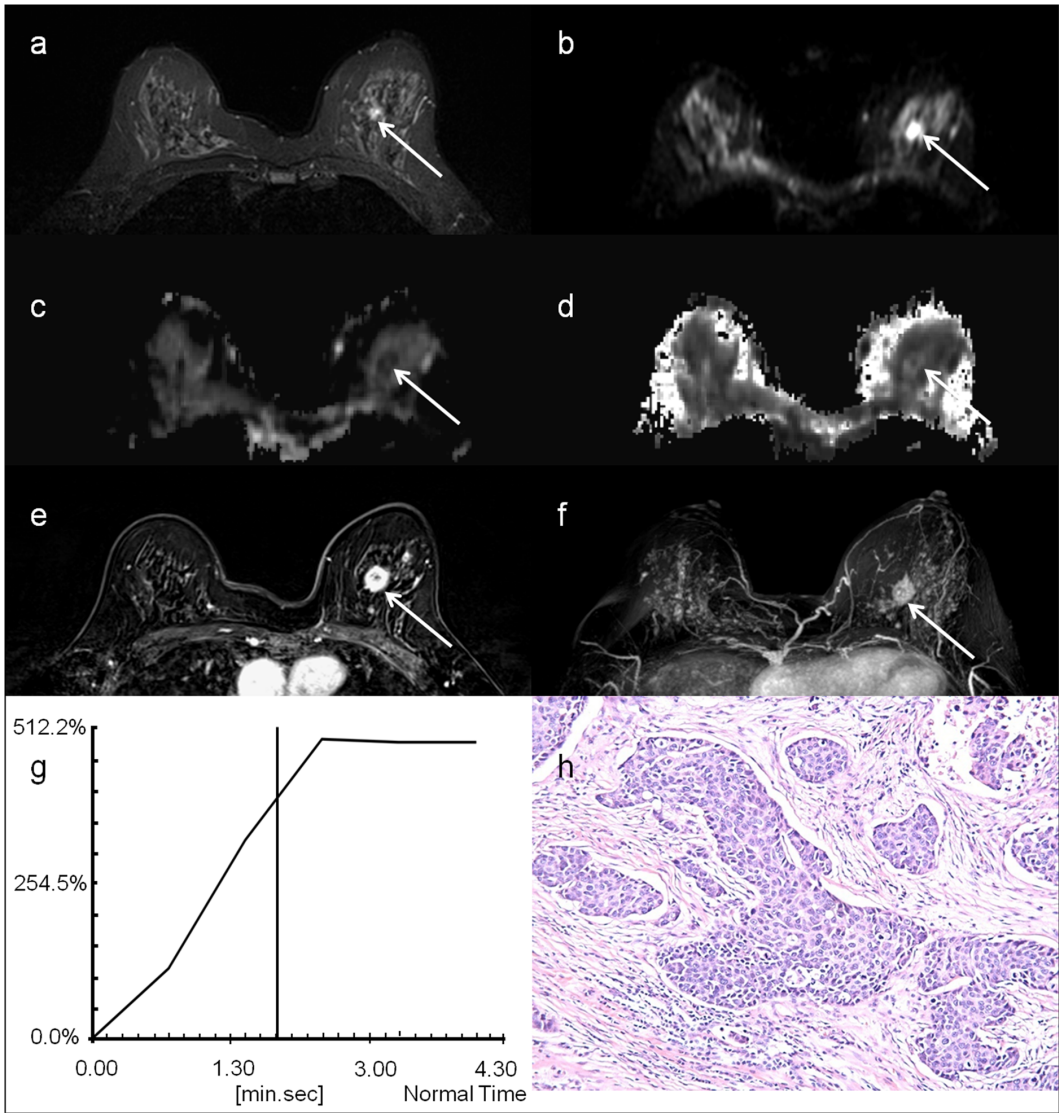
A 51 year-in-old woman with infiltrating ductal carcinomas, indicated by the white arrow: a) T2-weighted TSE image; b) DW image at b = 0; c) MD map; d) MK map; e) contrast enhancement map; f) MIP of enhance map; g) contrast enhancement curves and h) histological specimen.

Figure 2 shows the T2-weighted TSE images, b0 images, MD maps, MK maps, as well as the contrast enhancement maps, Maximum Intensity Projection (MIP) of enhancement maps, and contrast enhancement curves of a patient with benign breast tumor – fibroadenomas. Similarly, Figure 3 shows the T2-weighted TSE images, b0 images, MD maps, MK maps, as well as the contrast enhancement maps, MIP of enhancement maps, and contrast enhancement curves of a patient with malignant carcinoma – infiltrating ductal carcinomas.

**Figure 4:**
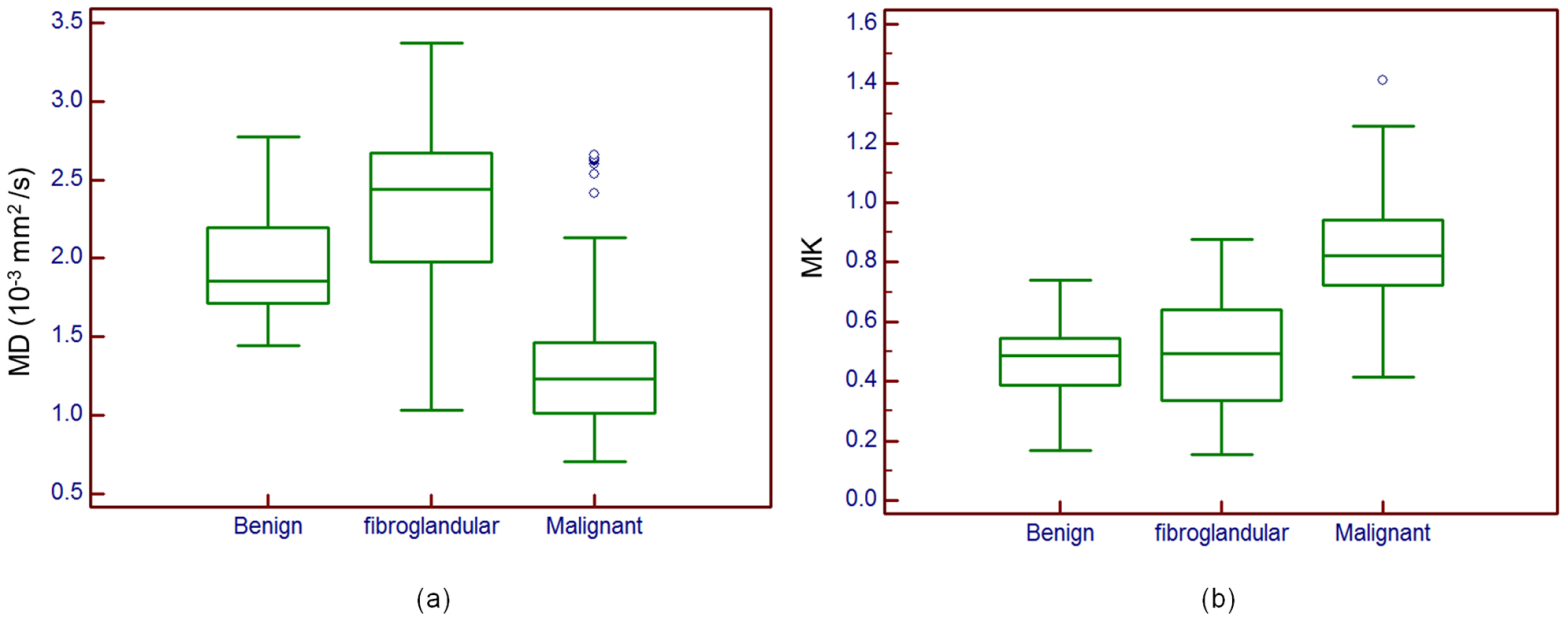
Box plot distribution: a) MD values for fibroglandular tissue, benign and malignant lesions; b) MK values for fibroglandular tissue, benign and malignant lesions. Outliers are also represented. Top and bottom of each box represent 25% and 75% percentiles of the MD and MK values, respectively. Horizontal line inside each box represents median value.

Figure 4 compares the MD and MK values in contralateral fibroglandular tissues, benign and malignant lesions. The MD values are 2.18±0.49 (10^−3^ mm^2^/s), 1.97±0.35 (10^−3^ mm^2^/s) and 1.20±0.31 (10^−3^ mm^2^/s), while MK values are 0.54±0.18, 0.47±0.14, 0.88±0.17 for contralateral fibroglandular tissues, benign and malignant lesions, respectively. The results reveals that both MD and MK values in fibroglandular tissue, benign and malignant lesions are significantly different (*P*<0.001).

**Figure 5:**
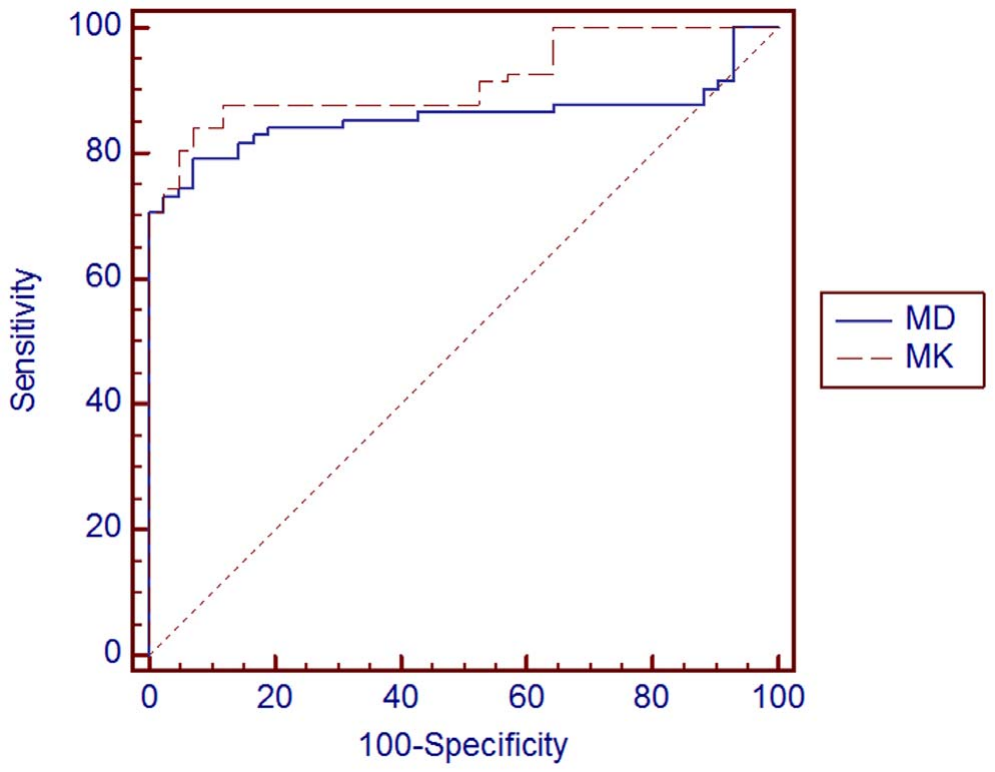
Receiver operating characteristic curve for MD and MK values used as predictors of malignancy in 124 breast lesions in 103 patients. Straight diagonal line spanning the middle of the graph indicates an AUC of 0.5.

Figure 5 shows the ROC curves for the evaluation of the ability of MD and MK values to differentiate between benign and malignant lesions. Results of ROC analysis are presented in Table 2. The area under curve (AUC) for MD was 0.86 with 95% confidence interval being 0.79–0.92. Using 1.58 (10^−3^ mm^2^/s) as MD cutoff value between benign and malignant lesions, the sensitivity was 79.3% and specificity was 92.9%; 20.7% were misclassified as benign, while 7.1% were misclassified as malignant. For MK, the AUC was 0.92 and the 95% confidence interval was 0.85–0.96. Using 0.69 as MK cutoff value between benign and malignant lesions, the sensitivity was 84.2% and specificity was 92.9%; 15.8%were misclassified as benign, while 7.1% were misclassified as malignant.

**Table 2:** Receiver operating characteristic (ROC) analysis for the accuracy of MD and MK in the diagnosis of breast lesions.

Parameters	AUC	Cutoff Value	Sensitivity	Specificity	*P* value
MD	0.86	1.58 (10^−3^ mm^2^/s)	79.3%	92.9%	<0.001
MK	0.92	0.69	84.2%	92.9%	<0.001

Note. – AUC  =  area under the curve; MD  =  Mean Diffusivity; MK  =  Mean Kurtosis.

## Discussion

Accurate diagnosis and classification of breast lesions have always been challenging using conventional mammography and ultrasound, especially in dense fibroglandular breasts [32]. Improved characterization of breast tumor type and degree of malignancy of breast cancer could greatly assist treatment planning and hence help improve patient's outcome. Diffusion MRI is a non-invasive functional imaging technique for providing valuable information on the water diffusion properties in tumor microenvironment. In particular, DKI can potentially augment conventional diffusion techniques for better breast lesion characterization. Recent studies have shown that DKI offers a method to evaluate the non-gaussian diffusion behavior in complex biological tissues in various brain diseases, including ischemic stroke, Parkinson diseases, traumatic brain injuries, and brain gliomas [27], [33]–[36]. The present study aimed to examine the capability of DKI in differentiating benign from malignant breast lesions.

Our study results demonstrate the potential utility of DKI for the characterization of breast lesions. The results of ROC analysis suggest that the use of MK lead to a higher sensitivity and specificity and lower percentage for misclassification than those of MD in determining benign and malignant breast lesions, indicating a better diagnostic performance, figure 5. Our study results also show that significantly higher MK values are found in malignant lesions, indicative of higher non-Gaussian diffusion, i.e., higher cellular complexity, than in benign lesions, as theoretically expected.

The behavior of DW signal with higher b-values, whether in benign or malignant lesions, is better characterized by DKI model rather than mono-exponential model, figure 1. It has been suggested that molecular motion of water becomes more restricted because of decreasing extracellular space as tumor cells proliferate and hence cellularity increases. This may reflect microstructural differences between benign and malignant tissues. Moreover, moderate correlation between water diffusivity and tumor cellularity has been illustrated previously. Changes in DKI parameters may reflect physiologic and morphologic alterations associated with breast tumor tissues. MK may be related to the degree of microstructural complexity. DKI may add valuable indications of microstructural changes to conventional diffusion techniques for the characterization of breast tumors, however, the mechanisms underlying the differences in MK alteration between benign tumors and malignant cancers have yet to be determined.

Although DCE-MRI using gadolinium chelates offers high sensitivity in detecting breast lesions and hence has been widely used for locating multiple small breast tumors, it suffers from low specificity in characterizing tumor tissues [37]. Indeed, certain benign breast lesions enhance in a similar fashion to malignant cancers [38], [39], which is confirmed in our study that the contrast enhancement curves for both benign and malignant lesions are in the same plateau pattern, figure 2 and 3. As a result, contrast enhancement is thought to be attributed to the proliferating activity of the hyperplastic or neoplastic parenchymal cells, in addition to the vessel density associated with tumor angiogenesis [40], [41]. Therefore, it is sometimes impossible to distinguish enhancing benign tumors from malignant carcinomas unambiguously based on the contrast enhancement patterns and morphologic features alone. Nonetheless, diffusion MRI could complement DCE-MRI in these circumstances [42]–[46]. In addition, the results of this study suggest that DKI is potentially a promising quantitative technique for improved characterization and evaluation of breast tumor lesions, and hence could greatly augment DCE-MRI. Note that the potential toxicity of gadolinium chelates limits the accessibility of DCE-MRI for patient with compromised kidney functions. In contrast, DKI does not require contrast agent administration and hence is more suitable for multiple longitudinal follow-up studies for monitoring tumor growth and response to therapy.

It is worthwhile to note that pronounced microvascular perfusion associated with tumor angiogenesis may lead to bi-exponential DW signal decay in breast tumor tissue [47], [48]. Indeed, MR signal attenuation in a voxel of highly perfused tissue is associated with the combined effects of molecular water diffusion and blood perfusion in the presence of magnetic field gradient [49], [50]. The contribution of micro-perfusion to the DW signal loss arises from intra-voxel spin dephasing due to pseudorandom motion of moving blood protons in the microvasculature, also known as intra-voxel incoherent motion [49]. Nevertheless, the blood microcirculation only dominates the pseudo-diffusion signal attenuation prominently at low b-values [8]. As such, we performed diffusion kurtosis analysis by fitting the DW signals with b-values starting from a non-zero b-value (250 s/mm^2^) to minimize the effects of pseudorandom vascular perfusion [51]–[53] in the current study.

Breasts consist of an exceptionally high content of fatty tissue. It has been shown that adipose tissue in breasts yields significantly lower MD value than normal fibroglandular tissue [54]. As a result, effective fat suppression is essential in breast DKI to minimize any partial volume effects from intravoxel fat signals [55]. It has also been suggested that the choice of fat suppression technique could influence the MD measurement, especially when large b-values are used, which is likely ascribed to the varying contribution from fat tissue to the DW signals [56], [57]. In the present study at 3T, we employed SPAIR, which utilizes adiabatic frequency-selective inversion pulses to invert and null fat signal, for fat suppression. Owing to compromised B1 homogeneity at high field, adiabatic inversion pulse that is insensitive to B1 inhomogeneity could suppress fat signal in breast effectively as shown in our study. Note that breast adipose tissue can be easily identified by exceptionally low signal intensity on non-DW b0 images, even they exhibit low MD value.

In our study, DKI was performed based on a single-shot spin-echo EPI sequence with 7 b-values in three orthogonal directions, and the MK was computed by averaging AKC over all directions. Indeed, the use of average AKC for the approximation of MK took an assumption of isotropic diffusion for simplicity, which might introduce the rotation variations and hence deteriorate the validity of MK in the cases of anisotropic diffusion. For improvement, a tensor could be used to describe the dependency of the measured diffusional kurtosis on the direction of the diffusion sensitizing gradients [23]. Nonetheless, the use of kurtosis tensor has been challenged for its lengthy acquisition time and complex postprocessing [58], involving at least 15 independent components of the tensor [23]. As such, rapid protocol and robust postprocessing approaches targeted for diffusion kurtosis tensor imaging remain necessary for the future DKI investigation.

Several potential limitations exist in the current study. First, we did not evaluate the influences of menopausal status and menstrual cycle on DKI parameters in breast tumor tissues. Slight variation in normal breast MD owing to hormonal fluctuations and hence water content throughout menstrual cycle has been reported previously [59]. Second, the findings in the current study were preliminary, a further multi-center study with large cohort is necessary. Further clinical evaluation on a larger patient population is warranted. Third, the non-linear squared fitting method used for DKI parameters computation in this study is susceptible to the noise for the low signal-to-noise data at high b-values. Therefore, advanced method such as multi-step weighted linear least squared (WLLS) approach [60], which could provide high performance in terms of accuracy/precision, is necessary. Fourth, histopathological evaluation is subject to both observer variation and variability based on the spatial focus of observation. Fifth, malignant lesions with central necrosis often show high ADC values in the area of necrosis and the rim of the lesion may be too thin for correct ROI placement. Nonfocal mass lesions as often seen in DCIS may not be categorized correctly with DWI even with a small ROI due to diffuse tumor spread and partial volume effects.

In conclusion, the results of this study suggest that DKI could provide valuable information on the diffusion properties related to tumor microenvironment by the simultaneous quantification of both MD and MK. DKI could improve tissue characterization of breast lesions and increase diagnostic confidence of breast tumors. Further studies with larger sample size are warranted to explore the full potential of DKI for non-invasive imaging of human breast lesions in clinical setting. Once fully validated, breast DKI may also serve as a non-contrast breast screening imaging technique, avoiding unnecessary biopsies.
